# Adjunctive intravenous then oral vitamin C for moderate and severe community-acquired pneumonia in hospitalized adults: feasibility of randomized controlled trial

**DOI:** 10.1038/s41598-023-37934-z

**Published:** 2023-07-23

**Authors:** Stephen T. Chambers, Malina Storer, Amy Scott-Thomas, Sandy Slow, Jonathan Williman, Michael Epton, David R. Murdoch, Sarah Metcalf, Anitra Carr, Heather Isenman, Michael Maze

**Affiliations:** 1 Department of Pathology and Biomedical Science, University of Otago, Christchurch, New Zealand; 2 Canterbury Respiratory Research Group, Canterbury District Health Board, Christchurch, New Zealand; 3 Department of Agricultural Sciences, Lincoln University, Lincoln, New Zealand; 4 Biostatistics and Computation Biology Unit, University of Otago, Christchurch, New Zealand; 5 Department of Infectious Diseases, Christchurch Hospital, Canterbury District Health Board, Christchurch, New Zealand; 6 Department of Medicine, University of Otago, Christchurch, New Zealand

**Keywords:** Outcomes research, Nutritional supplements

## Abstract

Patients hospitalised with community acquired pneumonia (CAP) have low peripheral blood vitamin C concentrations and limited antioxidant capacity. The feasibility of a trial of vitamin C supplementation to improve patient outcomes was assessed. Participants with moderate and severe CAP (CURB-65 ≥ 2) on intravenous antimicrobial treatment were randomised to either intravenous vitamin C (2.5 g 8 hourly) or placebo before switching to oral intervention (1 g tds) for 7 days when they were prescribed oral antimicrobial therapy. Of 344 patients screened 75 (22%) were randomised and analysed. The median age was 76 years, and 43 (57%) were male. In each group, one serious adverse event that was potentially intervention related occurred, and one subject discontinued treatment. Vitamin C concentrations were 226 µmol/L in the vitamin C group and 19 µmol/L in the placebo group (*p* < 0.001) after 3 intravneous doses. There were no signficant differences between the vitamin C and placebo groups for death within 28 days (0 vs. 2; *p* = 0.49), median length of stay (69 vs. 121 h; *p* = 0.07), time to clinical stability (22 vs. 49 h; *p* = 0.08), or readmission within 30 days (1 vs. 4; *p* = 0.22). The vitamin C doses given were safe, well tolerated and saturating. A randomised controlled trial to assess the efficacy of vitamin C in patients with CAP would require 932 participants (CURB-65 ≥ 2) to observe a difference in mortality and 200 participants to observe a difference with a composite endpoint such as mortality plus discharge after 7 days in hospital. These studies are feasible in a multicentre setting.

## Introduction

Administering supplemental vitamin C to treatment regimens for community acquired pneumonia (CAP) is an attractive option, but evidence of benefit is lacking^1,2^. Vitamin C cannot be synthesised by humans and concentrations in the peripheral blood are decreased in CAP^3–6^. Vitamin C is a potent antioxidant, able to scavenge a wide range of reactive oxygen species, thereby limiting tissue damage^7^. The vitamin also supports neutrophil migration to the infected site^5^, is a cofactor in the synthesis of noradrenaline and vasopressin^8^, and has other metabolic functions^9^. These observations have prompted investigation of the potential role of supplemental vitamin C therapy for CAP.

The published studies of the effect of vitamin C treatment on the outcome of CAP have been reviewed by the Cochrane collaboration, which identified one randomised controlled trial (N = 57)^1^. Those investigators included patients with acute bronchitis as well as CAP, and found that 200 mg of vitamin C administered orally was associated with fewer deaths (risk ratio 0.21 [95% CI 0.03, 1.66]), and a reduction in the symptom severity 2 and 4 weeks after admission^10^. Two other trials performed in children with pneumonia reported a decrease in duration of illness and length of hospitalisation^11,12^. Studies done in the intensive care setting of sepsis including pneumonia, have provided promising but not conclusive results^13,14^.

This study included patients with moderate and severe CAP. While the most secure endpoint to establish efficacy is death, the FDA has recommended an early clinical response of symptoms as an endpoint in clinical trials of CAP^15^. Others have demonstrated that comparison between treatments of proportions with an early clinical response (symptom improvement 72–120 h after the first dose of study drug) and time to clinical stability between treatments are essentially equivalent endpoints^16–18^. Both early clinical response and clinical stability have a high concordance with clinical success defined as survival with resolution or improvement of signs and symptoms of infection with no need for further antibacterial therapy.

This study was conducted to inform the design of a double-blind randomised controlled clinical trial embedded within routine ward based care in an acute tertiary care admitting hospital^19^. The study aims were to assess the availability of patients for recruitment and acceptability, dose and delivery of the intravenous administration of vitamin C, including the additional blood sampling, and follow-up procedures. The potential endpoints were the mortality rate, time to clinical stability, length of hospital stay, need for additional antimicrobial therapy, and readmission rate.

## Results

### Study recruitment

**Figure 1:**
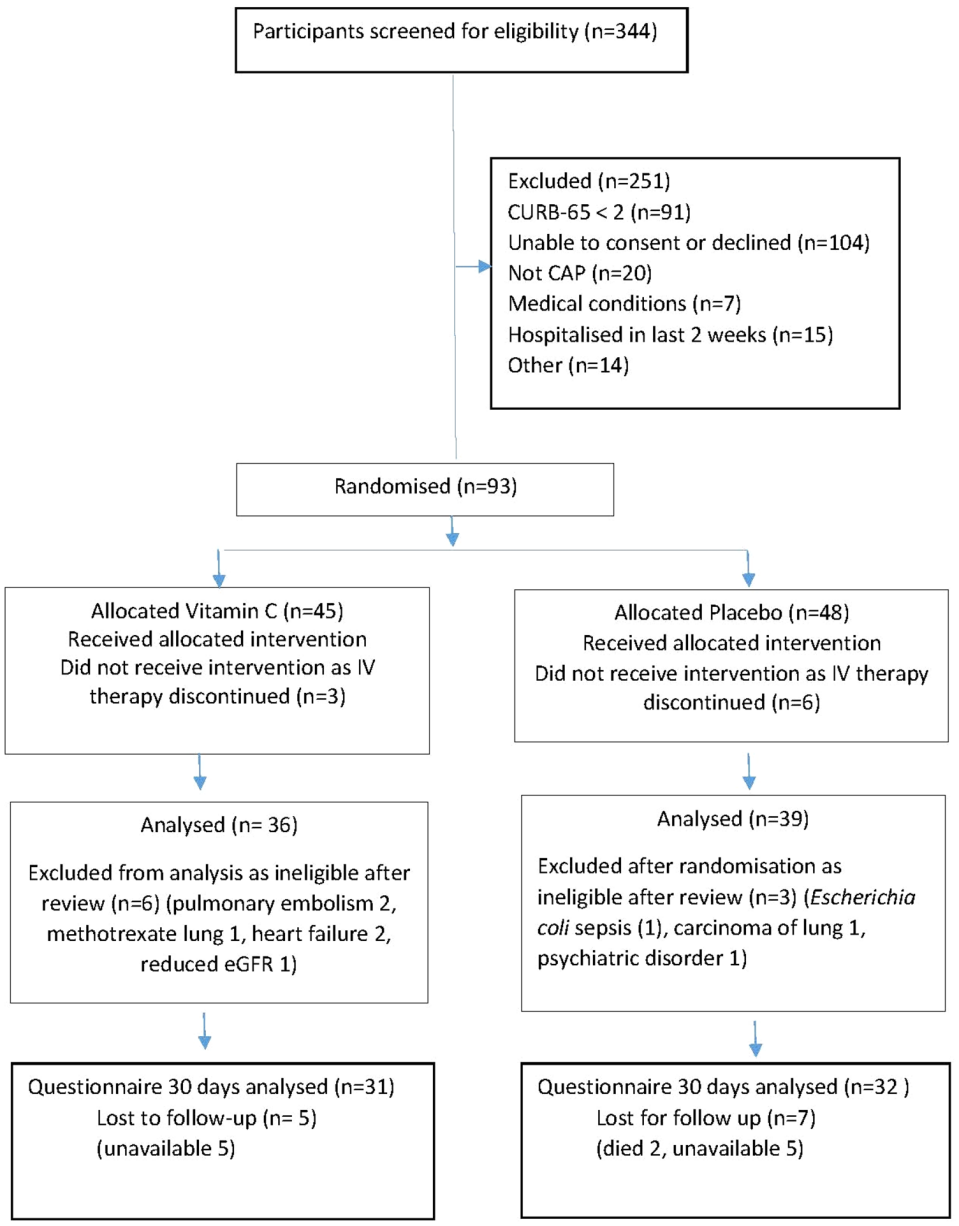
Participant flow.

Figure 1 shows the study recruitment and treatment diagram. Of 344 potential participants screened, 93 (27%) were eligible for inclusion. Enrolment was done on weekdays beginning on 25 November 2019 and continued until April 2021. Recruitment was suspended between 23 March 2020 and 13 May 2020 due to government mandated restrictions for the COVID-19 epidemic and for 8 weeks for staff leave. Over 55 weeks of active recruitment 93 subjects were randomised (1.7/week), but 9 were excluded for an alternative diagnosis and 9 had been changed to oral antimicrobial therapy before receiving the intervention. There were 101 exclusions prior to randomisation for confusion, dementia and refusal. Overall, 1.4 eligible patients per week were included.

Table 1 shows the baseline characteristics of the 75 patients included in the analysis. The median age at recruitment was 74.5 (range 31- 93) years and 43 (57%) were male. The baseline CURB65 pneumonia severity scores were similar between the two groups.

**Table 1:** Baseline characteristics of participants randomised to vitamin C or placebo.

Characteristics	Vitamin C (n = 36)	Placebo (n = 39)	Total (N = 75)
Demographics			
Age years, median (IQR)	74.5 (70, 83)	78.0 (70, 83)	76.0 (70, 83)
Female	21 (58%)	22 (56%)	32 (43%)
Ethnicity			
European	33 (92%)	35 (90%)	68 (91%)
Maori	1 (3%)	1 (3%)	2 (3%)
Pacific	0 (0%)	1 (3%)	1 (1%)
Asian	1 (3%)	2 (5%)	3 (4%)
MELAA	1 (3%)	0 (0%)	1 (1%)
Smoking status			
Never	11 (31%)	14 (36%)	25 (33%)
Past smoker	20 (56%)	18 (46%)	38 (51%)
Current smoker	3 (8%)	4 (10%)	7 (9%)
Unknown	2 (6%)	3 (8%)	5 (7%)
Consume alcohol weekly	15 (48%)	12 (40%)	27 (44%)
Co morbidities			
COPD	9 (26%)	13 (36%)	22 (31%)
History of chronic bronchitis, emphysema, bronchiectasis or cystic fibrosis	8 (22%)	11 (28%)	19 (25%)
Asthma	7 (21%)	9 (25%)	16 (23%)
Previous pneumonia	5 (15%)	8 (24%)	13 (19%)
Heart failure	3 (9%)	6 (17%)	9 (13%)
Cerebrovascular disease	5 (15%)	3 (9%)	8 (12%)
Renal disease	1 (3%)	3 (8%)	4 (6%)
Liver disease	1 (3%)	0 (0%)	1 (1%)
Diabetes	5 (14%)	8 (22%)	13 (18%)
Immune Suppression	1 (3%)	2 (6%)	3 (4%)
Other	4 (11%)	4 (11%)	8 (11%)
Antimicrobial therapy prior to admission			
Yes	5 (14%)	10 (26%)	15 (20%)
No	26 (74%)	26 (67%)	52 (70%)
Don’t know/not available	4 (11%)	3 (8%)	7 (9%)
Severity on admission			
CURB65 score, median (IQR)	2 (2, 3)	3 (2, 3)	2 (2, 3)
2	19 (53%)	19 (49%)	38 (51%)
3	14 (39%)	14 (36%)	28 (37%)
4	3 (8%)	6 (15%)	9 (12%)
Investigations at baseline			
Lobar consolidation	28 (80%)	28 (74%)	59 (77%)
Multilobar consolidation	7 (20%)	9 (24%)	16 (22%)
Pleural effusion	4 (11%)	7 (18%)	11 (15%)
CRP mg/L, median (IQR)^a^	130 (56–264)	158 (62–97)	148 (59–279)
Procalcitonin µg/L median (IQR)	0.34 (0.15–1.48)	0.421 (0.15–2.7)	0.41 (0.15–1.63
Creatinine µmol/L, median (IQR)	97 (83–110)	111 (89–19)	101 (89–19)
Estimated glomerular filtration rate mls/second, median IQR)	59 (46–69)	52 (45–60)	54 (45–66))
Haemoglobin concentration g/L, median (IQR)^b^	128 (118–135)	127 (118–139)	128 (119–137)
Total white blood count × 10^9^/L, median (IQR)^b^	14 (11–19)	12 (9.3–16)	13 (10–17)
Neutrophil count, × 10^9^/L, median (IQR)^b^	12 (8.1–16)	11 (7.3–14)	11 (7.7–15)
Microbiological diagnoses			
*Legionella* species	7*	6**	13 (17%)
*Streptococcus pneumoniae*	2	3	5 (7%)
Other	*Mycoplasma pneumoniae* (1), Alpha haemolytic streptococcus (1)	Influenza A (1), *Pneumocystis jiroveci* (1)	
No microorganism identified	25 (69%)	28 (72%)	53 (71%)

^a^Data missing for two participants in the vitamin C group.

^b^Data missing for one participant in the vitamin C group. * *L. longbeachae 5, L pneumophila 2,* ** *L. longbeachae 5, L micdadei 1.*

*MELAA* Middle Eastern, Latin American, African.

### Treatment delivery

The median (IQR) number of intravenous doses of vitamin C administered was 3 (3, 6) and for placebo was 6 (3, 7). All 75 subjects received their intravenous course in hospital and 22 completed their oral course in hospital. Two subjects discontinued the oral formulation in hospital because of adverse effects (see below). An intravenous dose of vitamin C was missed in one subject (3%) and 6 (15%) subjects missed a placebo intravenous dose. Two subjects (6%) missed an oral dose of vitamin C and 6 (16%) missed an oral dose of the placebo while in hospital. Fifty-one subjects were discharged to complete the course of treatment at home. Of those discharged with oral therapy, 29 (81%) were prescribed vitamin C and 22 (56%) placebo. At follow-up, 25 of 29 (86%) taking vitamin C and 20 of 22 (91%) taking placebo reported completing their course of treatment at home.

### Clinical outcomes

The clinical outcomes are shown in Table 2. There were two deaths of participants during their hospital admission (days 6 and 7 respectively) but no further deaths within 30 days. Both died from respiratory illness (CURB65 scores of 3 and 4) and were randomised to placebo. Six participants were admitted to ICU. The time in hospital, time from treatment initiation until discharge and time to clinical stability were suggestively, but not definitively shorter, in the vitamin C group. Sixty-three subjects (85% of 73 alive) completed the 30-day follow-up questionnaires. Of these 75% had some persisting symptoms attributed to the episode of CAP, and only 35% had returned to normal activities (Table 2).

**Table 2:** Outcome measures by treatment group.

Clinical outcomes	Vitamin C (n = 36)	Placebo (n = 39)	Total (n = 75)	*p* value
Died in hospital	0 (0%)	2 (5%)	2 (3%)	0.494
Died within 30 days of treatment initiation	0 (0%)	2 (5%)	2 (3%)	0.494
Died within 90 days of treatment initiation	0 (0%)	4 (10%)	4 (5%)	0.116
Admission to ICU	2 (6%)	4 (11%)	6 (8%)	0.675
Admission to discharge (hours) Median (IQR)	69 (48, 116)	121 (69, 179)	94 (56, 169)	0.071
Treatment to discharge (hours) Median (IQR)	48 (24, 98)	98 (46, 165)	73 (26, 144)	0.098
Treatment to clinical stability (hours) Median (IQR)	22 (-4, 90)	49 (18, 137)	41 (10, 98)	0.083
Plasma vitamin C after 24 h, N (%) (median IQR) ^a^	29 (81%) 226 (131, 461)	32 (82%) 19 (10, 31)	61(81%) 40 (17, 208)	< 0.0001
Follow-up				
Completed questionnaire N(%)^b^	31 (86%)	32 (86%)	63 (86.3%)	1
Readmission within 30 days^b^	1 (3%) (day 24)	4 (11%) (day 1 & 26)	5 (8%)	0.22
Readmission for CAP^b^	1 (2.7%)	1 (2.7%)	2 (2.7%)	1
Further antimicrobial therapy during follow-up^c^	7 (19%)	5 (14%)	12 (19.0%)	0.52
No persisting symptoms^c^	8 (22%)	10 (27%)	18 (29%)	0.64
Cough^c^	12 (33%)	10 (27%)	22 (35%)	0.57
Chest pain^c^	3 (8%)	5 (14%)	8 (13%)	0.51
Shortness of breath^c^	14 (39%)	15 (41%)	29 (46%)	0.89
Returned to normal activities^c^	12 (33%)	13 (35%)	25 (40%)	0.88
Fatigue^c^	20 (56%)	14 (38%)	34 (54%)	0.14

Unless otherwise stated values presented are the number of participants (%).

^a^Samples were not available for vitamin C analysis for day 1 for 7 participants in the vitamin C group, and 7 in the placebo group.

^b^The percentage was calculated after excluding those who had died.

^c^The percentage was calculated for replies to that question only.

### Acceptability and safety and of treatment

Of those who fulfilled the entry criteria only three patients (3%) who were eligible on screening (n = 96) declined to be enrolled after completing the consenting process. Of those who were consented and included all subjects completed the intravenous part of the study (100%). Two subjects stopped oral treatment in hospital for adverse events (see below). At follow-up, 25 of 29 (86%) self-reported taking vitamin C and 20 of 22 (91%) taking placebo to complete their course of treatment at home.

Thirty-one adverse events in the 30 days from randomisation were reported. There were 8 serious adverse events, including 2 deaths, and two readmissions for CAP (Table 2). The other serious adverse events were: hospital admission for deconditioning (1), gout (1), new diagnosis of GI malignancy (1), and fall with rib fracture (1). None were thought to be related to the study drug. Three adverse events were thought to be study medication related. Two had nausea and vomiting after an oral dose and stopped treatment (vitamin C 1, placebo 1). One subject disliked the oral medication flavour (placebo) but completed the course.

The other 20 adverse events reported were: prescription of further antibiotic therapy from their general practitioner (vitamin C 7, placebo 5); worsening of existing health conditions (vitamin C 2, diabetes control and exacerbation of COPD); deconditioning after admission to hospital (vitamin C 1, placebo 1); new episode of bronchitis (placebo 1) and urinary tract infection (vitamin C 1); nausea or vomiting after enrolment but prior to administration of the intervention (vitamin C 1, placebo 1).

### Plasma vitamin C concentrations

Baseline samples were available in 67 patients (89%). The median (IQR) baseline vitamin C concentrations were 15 (7, 25) µmol/L in the vitamin C group (31/36, 86%), and 16 (6, 27) µmol/L (36/39, 92%), in the placebo group. There was no difference between these groups (*p* = 0.93). Forty-one (60%) had deficient vitamin C concentrations (placebo 19, vitamin C 22) and 36 hypovitaminosis C (placebo 17 µmol/L, vitamin C 9 µmol/L). The plasma vitamin C concentrations (median, IQR) in samples taken 2–4 h after the third dose were higher in the vitamin C treated groups compared with the placebo group (Table 2).

## Discussion

The study was designed and powered as a feasibility study of supplemental vitamin C in moderate to severe CAP and provided some important insights on recruitment, acceptability, and clinical outcomes that will help design future studies.

Recruitment of patients was feasible as the percentage enrolled (27%) was similar to that expected (25%) but the recruitments rates were lower than expected (62 vs > 100 per year). This was attributable to several factors. The study was done during the COVID pandemic when there were strict lockdown periods in New Zealand. That resulted in major reductions in transmission of both COVID-19 and other respiratory pathogens^20–22^. Hospital based severe respiratory illness rates, including CAP, were well below the reference period (2015–2019) and there were dramatic reductions in detection of respiratory illness during and after lockdowns^23^. For example, laboratory-based surveillance systems reported a reduction of 67.7% in influenza during, and a 99.9% reduction after the lockdown^24^. Additionally, notification of cases from the general medical teams may have reduced recruitment of those admitted directly to the intensive care unit from the emergency department where there were competing trials on CAP being conducted. Patients who were confused and unable to consent were excluded which reduced expected recruitment. Overall, the COVID-19 epidemic was the most important influence on recruitment.

There was a high rate of acceptability, administration and delivery of the intravenous agents but there was a delay between admission and administration of the first dose of vitamin C (median 22 h) shortening the course of parenteral therapy. Ideally, adjunctive vitamin C should be administered concurrently with the antimicrobial therapy by randomising and treating patients in the emergency department. This would reduce the nadir of vitamin C concentration, replenish stores more quickly, and maximise any potential benefit of the vitamin C. The time course of action of vitamin C is not known but given the multiple metabolic benefits, this may be quite short. Seven intravenous doses were missed overall. Most of these occurred when subjects were transferred between the acute admission ward and the general medical wards or ICU. This problem was promptly eliminated once it was recognised. After discharge, patients self-administered the oral medication and 88% reported completing their course of treatment. The dose of vitamin C administered produced a rapid rise in the peripheral blood concentrations and these were about 12-fold higher in the treatment group compared with controls after one day and over three-fold higher than saturating levels (> 70 µmol/L), whereas the median concentration remained in the hypovitaminosis range in the placebo group^3^. Tissue levels were likely to have been restored, and antioxidant capacity replenished, but the plasma concentrations needed to have a measurable clinical benefit are not known. The doses used were safe with minimal adverse effects as reported previously^25–28^.

We did not identify a difference in our primary or secondary outcome measure between the groups. However, our study was not powered to show such differences and a benefit cannot be ruled out. The consistent signal towards a benefit from vitamin C across the measures including mortality, clinical stability, and length of hospital stay indicates that a definitive trial is warranted. The mortality in the control group of 5% (95% CI 1.4–17%) was lower than expected although still consistent with results from large multicentre data sets. Reports of case fatality ratios for CURB-65 score 2 of 9.2%, and CURB-65 > 2 of 22.3% give an estimated case fatality ratio in moderate/severe CAP of 16%^2^. Factors contributing to the low mortality include low infection rates with *S. pneumoniae* which is associated with severe CAP and death^2,29–31^. *S. pneumoniae* was detected in 7% of the diagnostic samples (blood, sputum and urine) from participants although there was no systematic effort to obtain diagnostic samples. Surveillance data from 26 countries, including New Zealand, demonstrated a 68% reduction in reported *S. pneumoniae* infections at 4 weeks and 82% at 8 weeks following the week population movements were restricted for COVID-19^28^. Secondly, exclusion of patients who were confused, which is an independent predictor of death from CAP, may have reduced the overall mortality^32^. Finally, patients admitted from the emergency department directly to the intensive care unit were not notified to the recruiting team as other trials were being conducted in the ICU. Overall, we think that the COVID pandemic had an important influence on both the numbers enrolled and on the mortality rate. A recent study of vitamin D supplementation in CAP done in our centre prior to the COVID epidemic requiring consent, and excluding patients who were confused, found a mortality rate of 11% in participants with a CURB-65 of ≥ 2 (11%)^33^. However, it would be preferable to include patients with confusion given the benign nature of the intervention. This would require ethical approval for provisional consent from family or their representatives as is provided for in legislation^34^.

Despite the low mortality there were fewer deaths in the vitamin C treatment group than the control group. This is a similar finding to that of Hunt et al*.* who reported the findings of a small RCT^10^. Combined data gives an estimate of likely benefit (supplementary Fig. 1) (random effects model: risk ratio = 0.19 (95% CI, 0.03–1.01) gives some support for designing a trial using mortality as an endpoint.

The endpoint for future trials needs careful consideration. All-cause mortality at 30 days death is a secure endpoint but would require a large multicentre study^32^. Assuming a mortality of 10% in the control group, and a risk ratio to be detected of 0.5, the main study would require a total sample size of 932 (80% power). Endpoints that demonstrate improved speed of recovery require fewer participants. For example, an analysis of the restricted mean time until discharge^35^, where those who die or are not discharged within seven days are given a value of seven days, would require a sample size of 200 to obtain 80% power to detect a difference of one day assuming a standard deviation of 2.5 days (supplementary Table 1). Other markers of clinical improvement such as readmissions, symptom scores or time to clinical stability as recommended by the FDA and others may also be suitable^16,33^. We did not identify an improvement in CAP symptoms unlike that reported by Hunt et al.^10^. Any effect on quality of life or resolution of specific CAP related symptoms after discharge would likely be small and would require very large studies to exclude any useful benefit.

The strength of this study has been the rigorous design and pragmatic execution within routine practice. Our study includes patients with less severe CAP who may benefit from vitamin C administration early in the clinical course when the pathophysiology is more reversable. Limitations include number of severe cases included, exclusion of those unable to consent, and the time to administer the first dose of intravenous vitamin C. The follow up data was limited by using self-reporting of adherence to study medication.

We conclude that dosing of vitamin C at 2.5 g IV Q8H followed by 1 g PO thrice daily was safe and well tolerated. The rise in vitamin C was saturating, and sufficient to achieve any expected benefits. Recruitment for a definitive trial would be feasible in a multi-centre study but would require approximately 930 participants to determine any effect on mortality, or 200 participants for a composite end-point.

## Materials and methods

### Study design and participants

The study was conducted in Christchurch Hospital, New Zealand, a secondary and tertiary care referral hospital serving more than 550,000 people. Participants were adults (aged ≥ 18 years) admitted to the general medical or respiratory services from November 2019 to April 2021 with CAP. This was defined as an acute illness acquired outside a health care setting with clinical features that included increased cough, sputum production, shortness of breath and feverishness, and a new inflammatory infiltrate on chest radiograph.

Potential participants were identified upon admission and notified to the investigators for possible enrolment. The exclusion criteria were: (1) admission to hospital > 48 h prior to screening, (2) unable to give informed consent, (3) CURB65 pneumonia severity score of < 2^2^, (4) pneumonia was not the principal reason for admission; (5) pneumonia associated with bronchial obstruction, (6) bronchiectasis or known tuberculosis; (7) hospital admission in the previous two weeks, (8) severe immunosuppression (e.g. neutropenia 350 cells/μL), or haematological malignancy, HIV positive and CD4 cell count below 350 cells/μL, currently receiving cancer chemotherapy, receiving prednisone > 20 mg daily or anti-rejection medication; (9) history of nephrolithiasis; (10) renal impairment (EGFR < 30 mls/sec, (11) G6PD deficiency, (12) haemochromatosis; (13) pregnancy or breast feeding.

Patients who were randomised, but subsequently found to have an alternative diagnosis, or were switched to oral therapy before receiving an intravenous dose of study agent were excluded from the study.

All clinical decisions were made by the clinical care team. Local guidelines that recommend dual therapy with a beta lactam antimicrobial agent (amoxicillin, amoxicillin/clavulanate or cefuroxime) and a macrolide (azithromycin) for all patients with CURB-65 score of ≥ 2 with some clinical discretion^2^.

The initial patient review, recruitment and consenting was done by one of the clinical team (S.T.C, M.M) after notification together with a research assistant. The clinical data was entered first into a paper form with pre-specified fields, and then into a RedCap database by the research assistant. Overall this required a morning for the clinician each day and a whole day for the research assistant for maintaining the database.

The study was approved by the New Zealand Northern B Health and Disability Ethics Committee (18/NTB/218) and all participants gave written informed consent. The trial was conducted according to the principles of the Declaration of Helsinki and was registered with the Universal trial number U1111-1222-3105 (20/02/2019) and Australia New Zealand Clinical Trials Registry, http://www.anzctr.org.au (ACTRN12619000256178).

### Intervention

The interventions (2.5 g vitamin C or placebo) was added to 100 ml of normal saline and administered over 20–30 min 8 hourly until the attending clinical team changed from intravenous antimicrobials to oral therapy. The initial supplier of the intravenous therapy was ASCOR L-500, McGuff Pharmaceuticals Inc, Santa Ana, CA, USA, but this became unavailable during lockdown and was changed to Sodium Ascorbate Solution (Biological Therapies, Braeside, VIC, Australia). The intravenous placebo was normal saline. Both the vitamin C and placebo tablets were sourced from Tishcon Corp, NY, USA and were identical in appearance.

### Randomisation and masking

The randomisation was done by the biostatistician (J.W.) by computer generated code (1:1 allocation) which was stored in the Christchurch Hospital Pharmacy. The process and dispensing of the intravenous and oral preparations were performed by Christchurch Hospital Pharmacy under the direction of the study biostatistician (J.W.) to ensure those recruiting, enrolling and assessing outcomes were blinded to treatment allocation. The interventions were transported and stored in identical opaque packaging and accessed and administered only by the nursing staff assigned to care for the patient in the ward. None of the research staff was responsible for administering the interventions.

### Study procedures

The participants baseline characteristics were obtained at enrolment included smoking, current medications and supplement use. The highest CURB-65 score (pneumonia severity) in the 12 h prior to enrolment was recorded^2,31^. Patient hospital records were reviewed and additional clinical data recorded including time and date of hospital admission and discharge, presenting features, past medical history, and chest radiograph reports recorded. Routine physiological, microbiological, haematological and biochemical laboratory results, length of stay, intensive care unit admission, antimicrobial therapy and readmission to hospital between study enrolment and the 30-day follow-up period were obtained from the computerised medical records. Date of death was from the national death register.

Adverse events were monitored by regular review of participants during hospitalisation. Reasons for missed doses of the study medication were taken from the electronic medication records (Medchart), electronic nursing notes, and interviewing subjects.

Thirty days after admission the investigators administered a brief questionnaire by telephone to record compliance with oral medication, adverse effects, persistence of symptoms from their presenting illness, and return to normal activity^36,37^.

Study data were collected and managed using REDCap electronic data capture tools hosted at University of Otago^38,39^.

Samples for plasma vitamin C concentrations (lithium heparin tubes) were retrieved from the refrigerated (− 20 °C) storage facility in the diagnostic laboratory within 6 h for baseline measures^40^. All subsequent samples for vitamin C analysis were collected on ice, separated within 30 min and stored at − 80 °C. All vitamin C analyses were measured after completion of the study to maintain blinding. The vitamin C concentrations were measured by high performance liquid chromatography as described previously^41^. Inadequate vitamin C status was defined as 24–50 µmol/L, hypovitaminosis 12–23 µmol/L and frank deficiency < 11 µmol/L. Routine laboratory tests were carried out at Canterbury Health Laboratories.

### Endpoints

The feasibility endpoints were:

1. Recruitment. Defined as the percentage of patients admitted to hospital with CURB-65 ≥ 2 CAP who were enrolled in the study.
2. Treatment delivery. Measured by the number of prescribed doses of intravenous and oral therapy in hospital and self-reported adherence of oral therapy after discharge.
3. Acceptability. This was defined as (a) the percentage of patients who fulfilled the entry criteria and consented into the study, (b) the percentage of patients who completed the intravenous treatment, (c) completed oral therapy, and (d) suffered no significant adverse events.
4. Adequacy of vitamin regimen. Determined by measurement of vitamin C concentrations at 24 h in comparison with placebo.

Endpoints proposed for the main study were: all-cause mortality was defined as death within 30 days of the index admission; length of hospital stay; time to clinical stability^17^; readmission to hospital within 30 days; resolution of respiratory symptoms; return to normal activity at 30 days; and need for further course of antibacterial therapy.

The time to clinical stability was defined as the time (hours) until all vital signs were stable for ≥ 24 h, or 24 h after discharge from hospital if clinical stability has not been reached at the time of discharge. Stable vital signs were temperature of 37.8 °C or lower, heart rate of 100 beats per min or lower, spontaneous respiratory rate of 24 breaths per min or lower, systolic blood pressure of 90 mm Hg or higher (≥ 100 mm Hg for patients diagnosed with hypertension) without vasopressor support, mental status back to level before occurrence of community-acquired pneumonia, ability for oral intake, and adequate oxygenation on room air (PaO_2_ ≥ 60 mm Hg or pulse oximetry ≥ 90%). The parameters were measured as part of clinical monitoring by the nursing staff who entered these electronically into the medical records. These were done a minimum of 6 hourly. All the data was reviewed in a blinded manner by and experienced clinician (STC).

### Sample size

There are an estimated 800 CAP admissions to Christchurch Hospital each year. Large prospective cohorts (n = 718 and 3233) report 50–55% of participants have moderate or severe CAP (CURB-65 of ≥ 2 or pneumonia severity index (PSI) > 3)^2,31^. It was assumed that if at least 25% of the eligible cases were recruited, enrolment rates could exceed 100 participants a year. This would provide sufficient information to estimate parameter means, variance, and proportions with acceptable precision.

### Statistical analysis

Recruitment was assessed by determining the number of patients approached during the study period, and the proportion who meet initial eligibility criteria, were randomised, were treated, and were ultimately deemed eligible for inclusion in the analysis. Randomised patients were not considered eligible for inclusion in the analysis if, upon review, it was discovered that pneumonia was not the primary diagnosis or the patient had changed to oral antimicrobial therapy before intravenous therapy could be given. Acceptability of the intervention and study processes were assessed by calculating the proportions of patients completing the prescribed course of treatment and follow-up questionnaires respectively.

Participant characteristics were summarised descriptively and tabulated by treatment group. Clinical outcomes of interest and treatment compliance were summarized as counts (percentages) for categorical variables, and median (interquartile ranges) for continuous time to event data. Between-group differences in outcomes by treatment group were explored using fisher’s exact tests for binary outcomes, or log-rank tests for time to event data censoring for death. All data was analysed using R (version 3.2.1 Vienna, Austria) statistical software. Two-sided statistical tests were used to generate p values and point estimates reported with 95% confidence intervals^42^.

### Institutional review board statement

The study was conducted in accordance with the Declaration of Helsinki, and approved by the Health and Disability Ethics Committee (18/NTB/218, approved 13 March 2019).

### Informed consent

Informed consent was obtained from all subjects involved in the study. Informed consent has been obtained from the patient(s) to publish data from the study.

## Supplementary Information

Supplementary Information 1.

Supplementary Information 2.

## Data Availability

The data presented in this study are available on request from the corresponding author. The data are not publicly available and stored on a secure system at the University of Otago, Christchurch.
