# Supplementary material for: Adjunctive intravenous then oral vitamin C for moderate and severe community-acquired pneumonia in hospitalized adults: feasibility of randomized controlled trial
Source: Sci Rep. 2023 Jul 23;13:11879. doi: 10.1038/s41598-023-37934-z (PMC10363531; doi:10.1038/s41598-023-37934-z)

Adjunctive Intravenous then Oral Vitamin C for Moderate and Severe Community-Acquired Pneumonia in Hospitalized Adults: Feasibility of Randomized Controlled Trial

Stephen T. Chambers[1*], Malina Storer[2], Amy Scott-Thomas[1], Sandy Slow[3,1], Jonathan Williman[4], Michael Epton[2], David R. Murdoch[1], Sarah Metcalf[5], Anitra Carr[1], Heather Isenman[5] and Michael Maze[2,6].

Supplementary figure 1 – Random effects model.

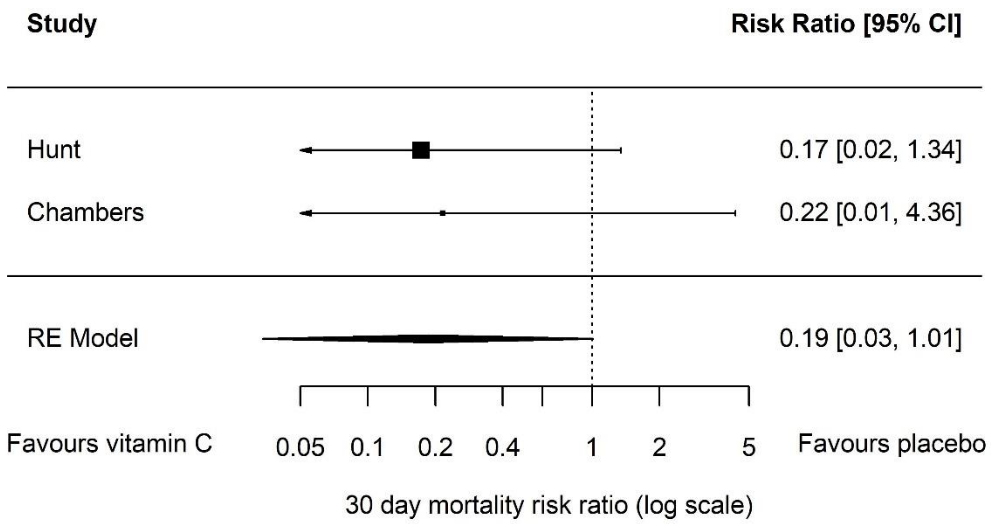

Supplement: Supplementary file 1 — Supplementary Information 1. [file 41598_2023_37934_MOESM1_ESM.pdf]
