# Supplementary material for: Adjunctive intravenous then oral vitamin C for moderate and severe community-acquired pneumonia in hospitalized adults: feasibility of randomized controlled trial
Source: Sci Rep. 2023 Jul 23;13:11879. doi: 10.1038/s41598-023-37934-z (PMC10363531; doi:10.1038/s41598-023-37934-z)
Supplement: Supplementary file 2 — Supplementary Information 2. [file 41598_2023_37934_MOESM2_ESM.pdf]

# Adjunctive Intravenous then Oral Vitamin C for Moderate and Sever Community-Acquired Pneumonia in Hospitalized Adults: Feasibility of Randomized Controlled Trial

**Stephen T. Chambers[1*], Malina Storer[2], Amy Scott-Thomas[1], Sandy Slow[3,1], Jonathan Williman[4], Michael Epton[2], David R. Murdoch[1], Sarah Metcalf[5], Anitra Carr[1], Heather Isenman[5] and Michael Maze[2,6].**

**Supplementary table 1**

Comparison of restricted mean time until discharge or clinical stability by treatment group. Those did not achieve the outcome within seven days, or who died within seven days, are given a value of 7 days.

| **Days until event, mean (sd) [range]** | **Vitamin C** | **Placebo** | **Total** | **p value** |
|---|---|---|---|---|
| **Restricted mean difference**. | N = 36 | N = 39 | N = 75 | |
| Admission to discharge | 3.6 (1.9) [0.9 - 7.0] | 4.7 (2.0) [1.1 - 7.0] | 4.2 (2.1) [0.9 - 7.0] | 0.02 |
| Admission to clinical stability | 2.7 (2.3) [0.1 - 7.0] | 3.6 (2.3) [0.1 - 7.0] | 3.2 (2.3) [0.1 - 7.0] | 0.11 |
| Treatment to clinical stability | 2.9 (2.12) [0.1 - 7.0] | 4.0 (2.4) [0.1 - 7.0] | 3.5 (2.34) [0.1 - 7.0] | 0.039 |

P values calculated by t-test
